# Dually nanocoated planar waveguides towards multi-parameter sensing

**DOI:** 10.1038/s41598-021-83324-8

**Published:** 2021-02-11

**Authors:** Ismel Dominguez, Ignacio Del Villar, Omar Fuentes, Jesus M. Corres, Ignacio R. Matias

**Affiliations:** 1 Institute of Smart Cities, Public University of Navarre, 31006 Pamplona, Spain; 2 Department of Telecommunications and Electronics, Pinar del Río University, CP 20100 Pinar del Río, Cuba; 3 Department of Electrical and Electronic Engineering, Public University of Navarre, 31006 Pamplona, Spain

**Keywords:** Optical sensors, Nanophotonics and plasmonics

## Abstract

The incidence of light on the edge of a glass coverslip for a microscope slide, deposited with a thin film on both faces, permits exciting two resonances in each polarisation state of the input light, TE and TM. This dually nanocoated waveguide can be used for detecting simultaneously two different parameters on the basis of a further deposition of suitable materials on each face. As an example, the possibility of detecting temperature and humidity by using polydimethylsiloxane and agarose coatings, respectively, was demonstrated, which opens the path for the development of other dual-parameter sensors, and for even more parameters in cases in which each face of the coverslip is patterned. Moreover, the device was optimised in order to position two resonances in the near infrared (NIR) and two resonances in the visible region, with sensitivities of 0.34 nm/°C and 0.23 nm/%RH in the visible region and 1.16 nm/°C and 0.34 nm/%RH in the NIR, respectively, demonstrating the possibility of using the device in both spectral ranges and opening the path for the development of sensors based on multiple resonances, each one related to a different parameter to be detected.

## Introduction

In the domain of optical sensors, there exist multiple configurations which exploit different phenomena. Perhaps the three most extended platforms are surface plasmon resonance (SPR), based on the Kretschmann configuration, optical fibres, and whispering gallery mode resonators^1^. One of the main topics these platforms try to address is the ability to detect several parameters with the same device^2–4^. However, none of them present the capability of a recently published platform that consists of lateral incidence of light on the edge conventional planar waveguides, such as glass slides or coverslips^5^; to use both sides of the waveguide. This is something not possible even with its closest counterpart, the SPR based on the Kretschmann configuration, which typically employs a glass slide coated on the upper or the lower face, whilst the other face is fixed to a coupler prism^6^. This is true because the lateral incidence configuration exploits lossy modes^7,8^, also named guided modes in the literature^9^, a phenomenon quite different from surface plasmons. While surface plasmons resonances (SPRs) occur when the real part of the thin-film permittivity is negative and higher in magnitude than both its own imaginary part and the permittivity of the material surrounding the thin film, lossy mode resonances (LMRs) are generated when the real part of the thin-film permittivity is positive and higher in magnitude than both its own imaginary part and the material surrounding the thin film^10^.

**Figure 1:**
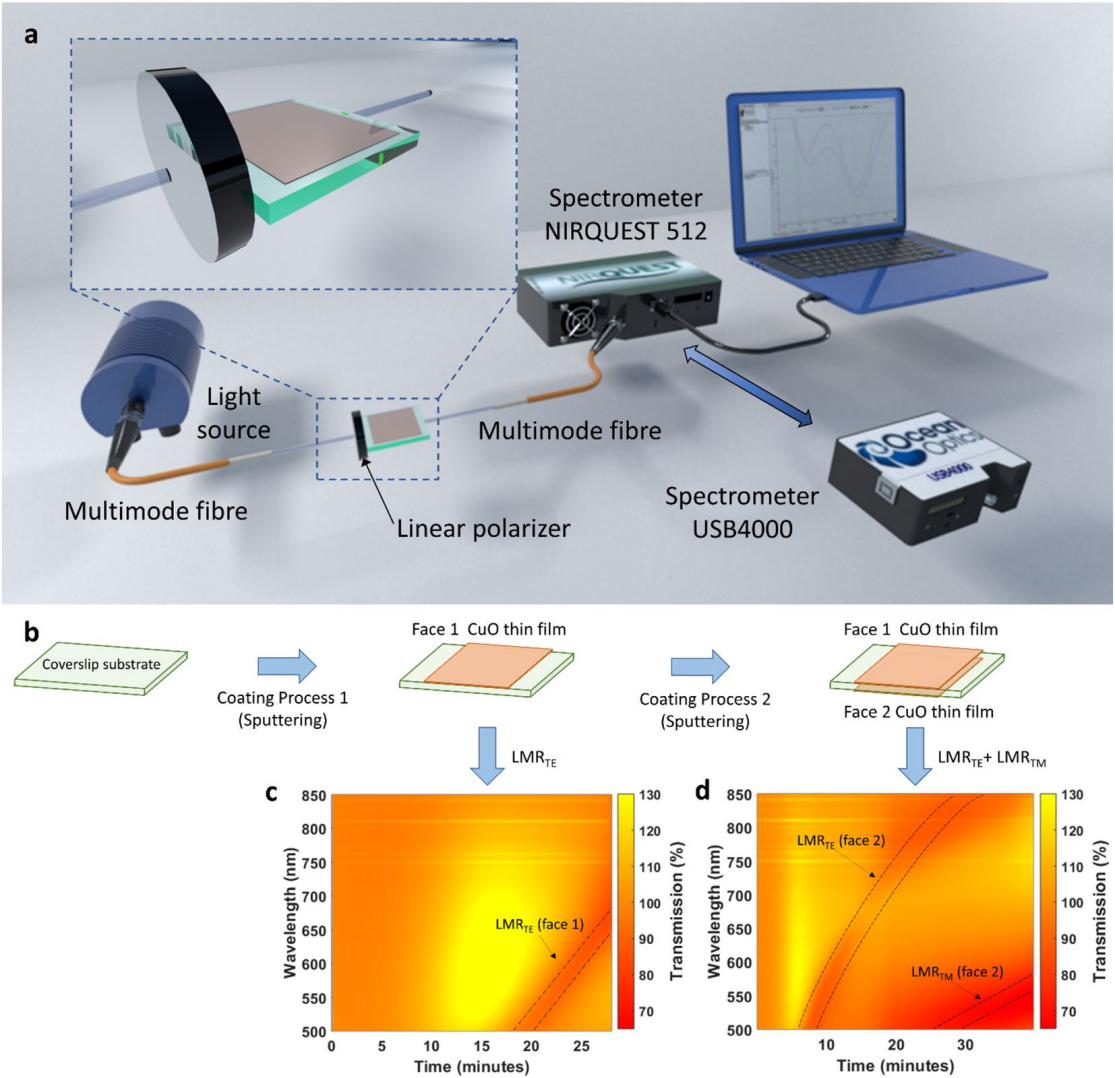
Fabrication and characterization of the sensor. (**a**) Experimental setup where a coverslip for a microscope slide is excited with light from a broadband source. (**b**) Both the upper face (face 1) and the lower face (face 2) of the coverslip are deposited with a CuO thin film. (**c**) Colour map representing the transmitted light as a function of time during the deposition of a CuO thin film on face 1 of the coverslip. (**d**) Colour map representing the transmitted light as a function of time during the deposition of a CuO thin film on face 2 of the coverslip.

As a result of the different excitation conditions for LMRs compared to SPRs, the best angles of incidence for LMR generation are those approaching 90°^11^, whereas SPRs are typically obtained with angles ranging from 40 to 75°^12,13^. This is what makes possible the lateral incidence of light on the edge of the coverslip^5,14^ (see Fig. 1a). This enables the use of the upper and lower faces for sensing two different parameters. However, in order to make this possible it is necessary to exploit another major advantage of LMRs compared to SPRs, i.e., the ability to progressively shift the position of the LMR in the vis/NIR spectrum by increasing the thickness, whilst new LMRs, related to new lossy modes guided by thin film, are positioned in the optical spectrum^15^. This simple control, which permits locating the LMRs in separate wavelength ranges in the optical spectrum, is not possible with SPR-based devices, which typically generate in a specific wavelength range one resonance that disappears when the thickness of the coating reaches a certain value^16^.

Thanks to the simplicity of generating multiple resonances with LMR-based devices, along with the capability to deposit suitable materials on both the upper and the lower faces of the coverslips, it was possible to create a dually nanocoated planar waveguide for sensing temperature and humidity. To this purpose, both faces of the coverslip were deposited, initially, with a single material that adequately induced the resonances. After that, one face was deposited with a second coating specifically sensitive to temperature, whereas the other face was coated with a material that is specifically sensitive to humidity. This is a proof of concept that could be easily extended to other applications, such as dual-parameter detection of gases or even dual-parameter chemical or biological detection in a microfluidic system.

## Results and discussion

### Generation of spectra with dual resonances

One of the main problems to control in a dually nanocoated planar waveguide is that the two resonances generated will overlap each other if they are not appropriately positioned. Therefore, a careful design must be performed, in which the position of the resonance after the first coating on both faces of the planar waveguide must be considered along with the further shift induced by the deposition of the second coating.

For the sake of simplicity, henceforward the two faces of the coverslip that will be deposited will be called face 1 and face 2, assigned arbitrarily to equivalent faces (see Fig. 1b).

In Fig. 1c the evolution of the optical spectrum is shown as a function of time during the deposition of the first coating, copper oxide (CuO), on face 1 of the coverslip. After 20 min of deposition, there is a band that shifts from 500 to 670 nm and that, according to the literature, is the first LMR, generated by the guidance of a TE lossy mode in the coating and consequently called LMR_TE_^10^.

After this deposition, face 2 of the coverslip was also deposited with CuO (see Fig. 1d). Here, it must be highlighted that a new reference was taken in order to avoid the presence of the TE resonance of the first coating in the spectrum, which permits observing, without any interference, the generation of the TE resonance obtained with the CuO coating deposited on face 2. In addition to this, the LMR_TM_ of face 2 is visible at short wavelengths in the optical spectrum (see Fig. 1d).

**Figure 2:**
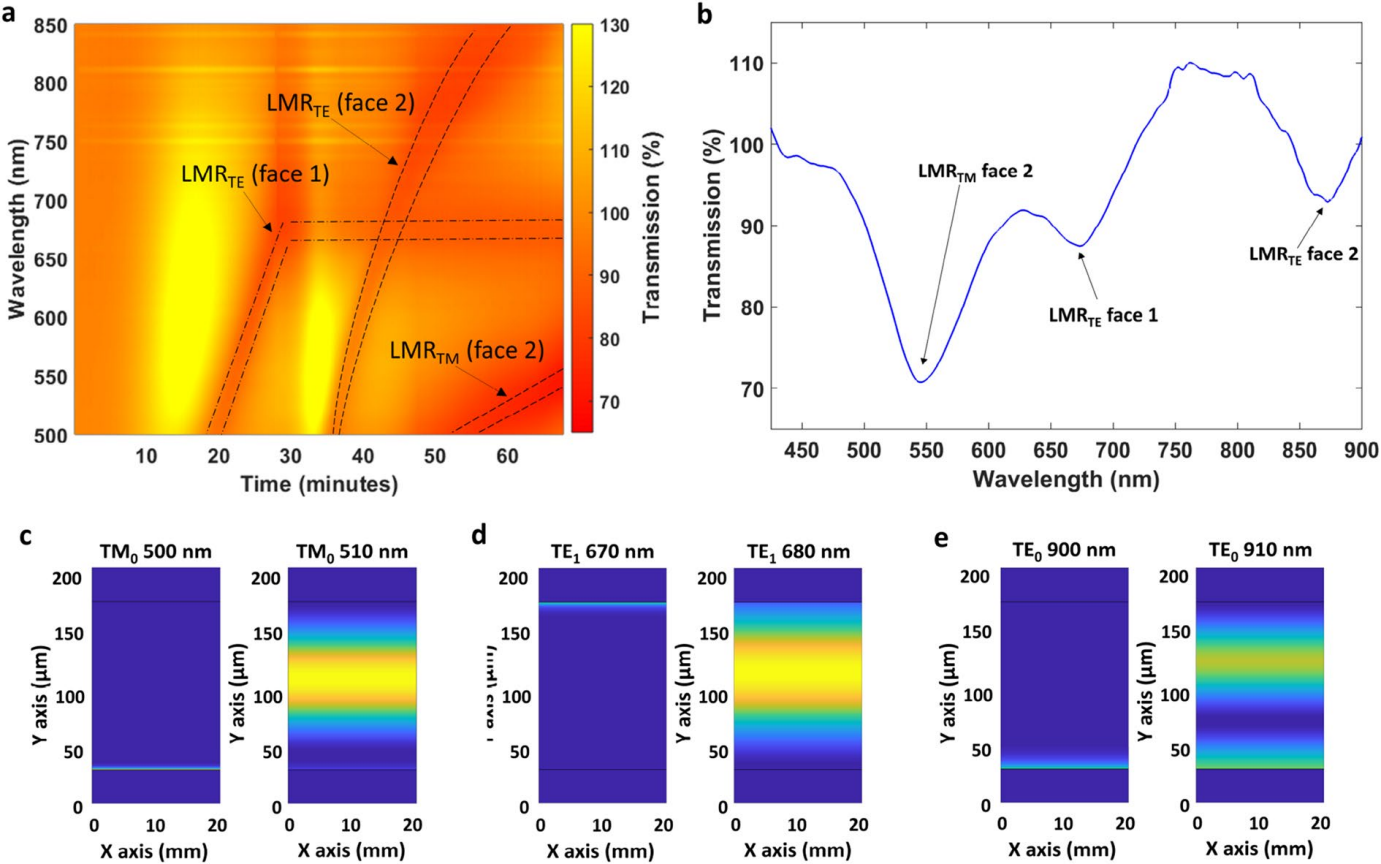
(**a**) Colour map representing the transmitted light as a function of time during the deposition of CuO on both faces of the coverslip. (**b**) Final optical spectrum in the visible range after deposition of CuO on both faces of the coverslip. (**c–e**) Optical field intensity distribution of TM_0_, TE_1_ and TE_0_ in the cross-section of the coverslip at the wavelength ranges where the transition to guidance of these modes takes place.

Figure 2a shows the complete deposition process of both faces of the coverslip, where the first thirty minutes show the same information presented in Fig. 1c, whereas this is not the case if we compare the second thirty minutes of Fig. 2a with Fig. 1d, because in Fig. 2a the reference was taken before starting the deposition on face 1 of the coverslip. As a result, if we compare Fig. 1d with the second thirty minutes of Fig. 2a, in Fig. 2a there is a clear and constant band located at 670 nm, which is the LMR generated with the coating deposited on face 1 of the coverslip.

The final spectrum after the deposition on both faces of the coverslip is shown in Fig. 2b, where the three LMRs are observed at different wavelength ranges: from 450 to 500 and from 850 to 900 nm the LMR_TE_ and the LMR_TM_ corresponding to face 2, and from 650 to 700 nm the LMR_TE_ corresponding to face 1.

In addition, the structure was analysed with FIMMWAVE (the parameters are shown in Methods section), which permitted to observe the transition to guidance of TM_0_, TE_0_ and TE_1_ modes at these wavelength ranges (Fig. 2c–e). A more detailed analysis is shown in Fig. S1–S3 in the supplementary material, whilst the effective indices of the first 10 TM and the first 10 TE modes are represented in Fig. S4.

**Figure 3:**
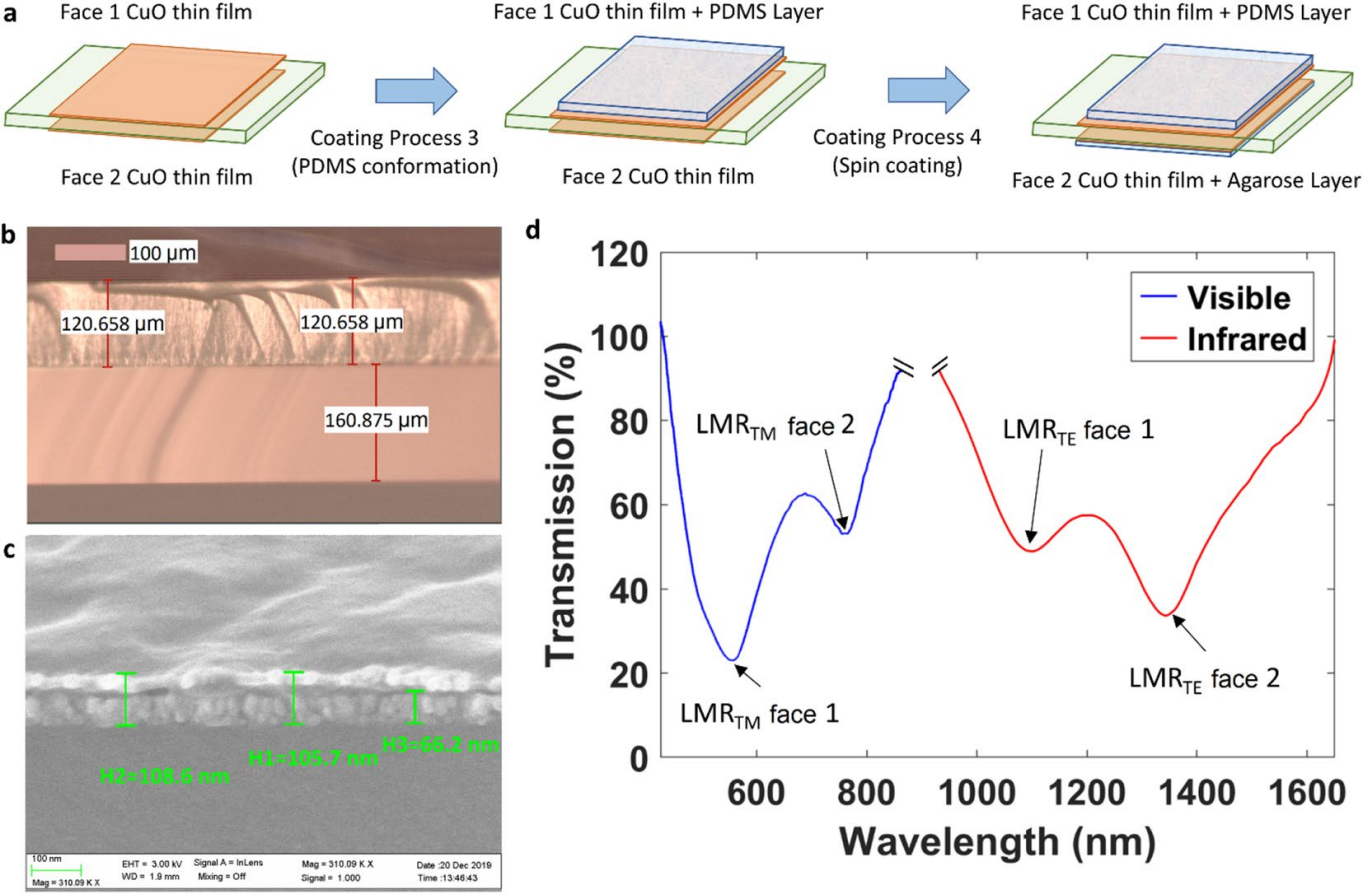
Deposition and characterization of agarose and PDMS coatings. (**a**) The upper face (face 1) and the lower face (face 2) of the coverslip are respectively deposited with agarose and PDMS. (**b**) Inverted microscope image showing the thickness of the PDMS coating, around 120 μm, deposited on a coverslip of thickness around 160 μm. (**c**) SEM microscope image showing the thickness of the agarose coating deposited on face 2 of the coverslip. The agarose layer can be distinguished on top of the CuO layer. The overall thickness of both layers is around 100 nm. (**d**) Optical spectra in the visible and NIR ranges after deposition of agarose and PDMS. Four LMRs are observed: two in the visible region, both of them TMs, and two in the NIR region, both of them TEs.

In order to obtain the final sensor that detects two parameters, two materials with high sensitivity to temperature and humidity were selected for deposition on face 1 and 2 of the sensor: PDMS and agarose, respectively^17,18^. The deposition process is described in Fig. 3a and the thickness of the coatings was characterized in Fig. 3b,c. The PDMS coating thickness was around 120 μm (see Fig. 3b), several order of magnitude thicker than the CuO coating, of the order of tens of nm. Therefore, it was not possible to visualize the CuO layer, though CuO was deposited under the same conditions in another coverslip and the thickness was 36 nm. Regarding the agarose layer, it was much thinner than the PDMS layer. Consequently, in Fig. 3c both the CuO and the agarose coatings can be distinguished. The overall thickness of both layers is around 100 nm and the thickness of the CuO is 66 nm.

Unhopefully, after the deposition of PDMS on face 1 of the coverslip and agarose on face 2, there was an overlap of the LMRs. This was due to the higher wavelength shift induced by PDMS compared to agarose (400 nm vs 300 nm). Therefore, a new sensor was fabricated in which the LMRs corresponding to face 2 were located at a longer wavelengths. With this new design, it was possible to solve the issue of the previous sensor. It is important to indicate that the basis for the optimization was not the thickness. The sensor was optimised by tracking the LMR while it was being deposited. Considering that the wavelength shift induced by the deposition of agarose and PDMS, the deposition process of CuO on each face of the coverslip was stopped when the corresponding LMR was located at a wavelength that permitted to obtain two separated LMRs in the infrared region, one for agarose and one for PDMS. In addition, due to the high refractive index of CuO, a good separation is obtained also in the visible region for the two LMRs obtained at TM polarisation, one corresponding to the face deposited with agarose and the other to the face deposited with PDMS.

Figure 3d shows, after deposition of PDMS and agarose, the two LMR_TE_ bands corresponding to each face of the coverslip in the NIR spectral region and the two LMR_TM_ bands corresponding to each face of the coverslip in the visible spectral region.

In addition to the question of optimising the design so that the different LMRs are separated from each other after the deposition of PDMS and agarose, there is a major reason for positioning the LMRs generated with face 1 at shorter wavelengths than the LMRs generated with face 2. Face 1 is used for sensing temperature on the basis of a PDMS coating, and it is well known from the literature that the refractive index of PDMS decreases as a function of temperature^17,19^. On the other hand, face 2 is used for sensing humidity on the basis of an agarose coating; and it is well known that agarose increases its refractive index as a function of humidity, due to the absorbance of water^18,20^. Therefore, the LMR corresponding to the face deposited with PDMS is shifted to shorter wavelengths as the temperature increases, whereas the LMR corresponding to the face deposited with agarose is shifted to longer wavelengths. In this way, overlapping of both resonances during the tests at different temperature and humidity is avoided, as will be shown in the next subsection.

### Characterisation of the dually nanocoated planar waveguide sensor

Henceforward, the experiments were performed in the climatic chamber described in the Methods section. The sensor was tested at different temperatures ranging from 30 to 70 °C with a constant humidity of 30%RH (see Figs. S5, S6 and S7). The results show that the LMRs induced by the coating deposited on face 1, both in the NIR and in the visible region, shift to shorter wavelengths as the temperature increases, whereas the opposite is true when the temperature decreases. This occurs because PDMS was deposited on face 1. Conversely, the LMRs induced by the coating deposited on face 2, on which an additional agarose layer was deposited, show no perceptible sensitivity to temperature.

**Figure 4:**
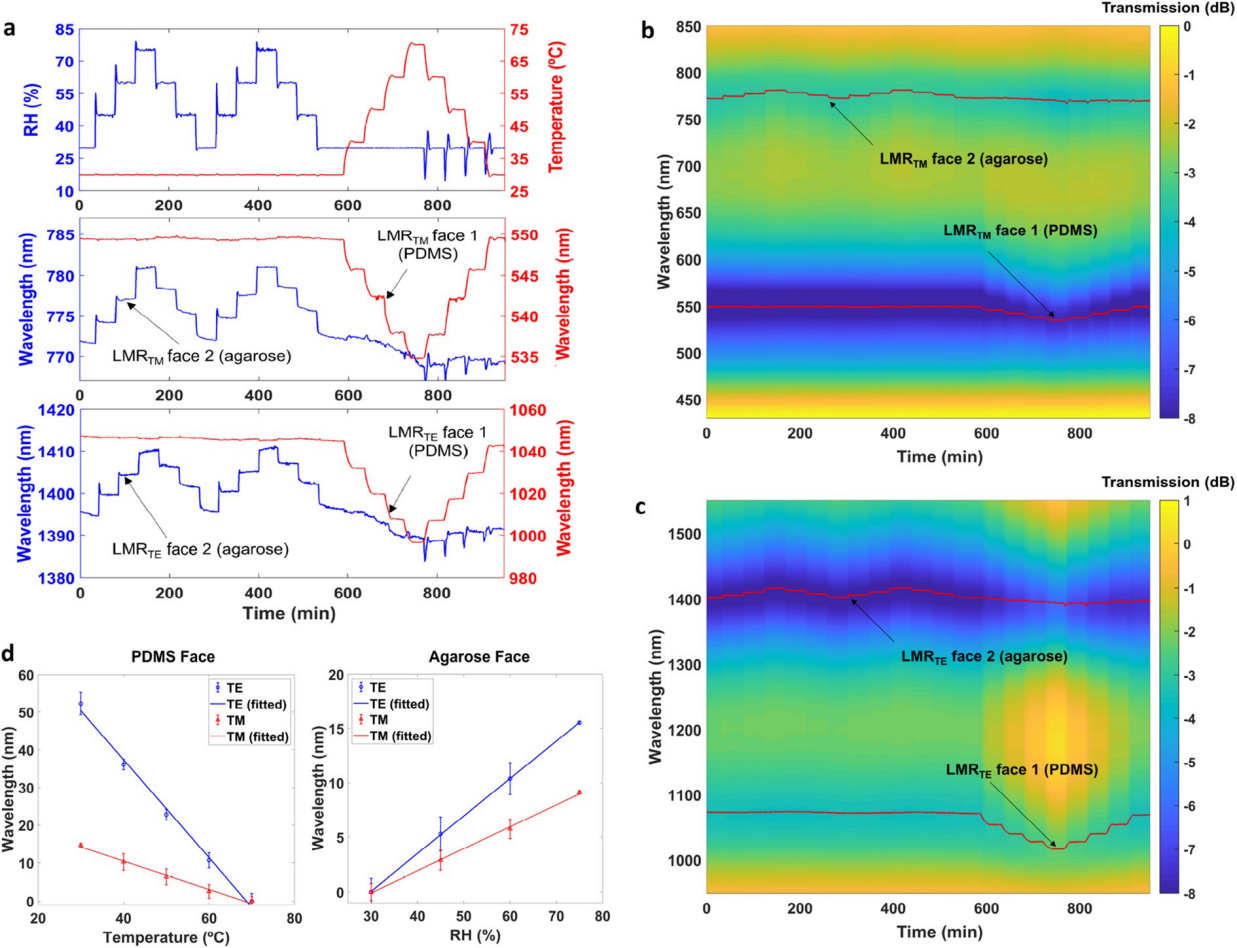
Humidity and temperature response of the sensor. (**a**) Humidity and temperature values registered by the electronic sensor compared with the wavelength shift in the visible and in the NIR range of the LMR_TM_ and LMR_TE_ generated by face 2, coated with agarose, and of the LMR_TM_ and LMR_TE_ generated by face 1, coated with PDMS. (**b**) Spectral evolution in the visible range of the optical sensor. (**c**) Spectral evolution in the NIR range of the optical sensor. (**d**) Relative wavelength shift as a function of temperature and humidity for both LMR_TE_ (NIR) and LMR_TM_ (visible), induced by face 1 (PDMS) and by face 2 (agarose).

After the temperature analysis, a test of humidity was performed in the same experiment. Figure 4a compares the results of the electronic temperature and humidity sensors of the climatic chamber with the results of the optical sensor in the visible region and in the NIR region. The response time is similar to the sensors of the climatic chamber, which indicates that the optical sensor is equal or better than the sensor of the chamber in terms of response time. Regarding the decline of the agarose blue line after 600 min, it is due to the loss of water in the thin-film due to the previous time segments at temperatures above room temperature.

In addition, the colour map in Fig. 4b,c permits an analysis of the transmitted power at all wavelengths, i.e., the evolution of the optical spectrum. The lines in red, representing the central wavelength of the LMR, follow the blue regions corresponding to the LMR attenuation bands. However, the results of Fig. 4b,c permit the observation that, in addition to the wavelength shift of the bands, there is also variation of the depth of the resonance. For instance, when the LMRs are more separated from each other, e.g., during the time interval from 700 to 800 min, the depth of the LMR located at 500–550 nm diminishes, probably because the other LMR has less influence on it. However, in terms of the LMR central wavelength, this effect was not observed in Fig. 4a because the central wavelength is ruled by the cut-off condition of each LMR^10^, which depends on the coating deposited on each face of the coverslip.

In order to analyse the sensitivity, in Fig. 4d those LMRs sensitive to temperature and humidity are evaluated as a function of the different temperature and humidity values studied in Fig. 4a–c. The results represent 4 up and down cycles of humidity and 4 up and down cycles of temperature. Therefore, this means 5 and 4 repetition times for the extreme points in the graph respectively, 30% and 75% of relative humidity, and 8 points for 45 and 60% relative humidity. Regarding temperature, 5 and 4 repetition times were performed for the extreme points of 30 and 70 °C and 8 points for 40, 50 and 60 °C. It is important to note that the humidity sensor presents hysteresis, which explains the big error bar for 45 and 60% humidity. In addition, the average sensitivity in the temperature range from 30 to 70 °C is 0.34 nm/°C in the visible region and 1.16 nm/°C in the NIR region, whereas the sensitivity to humidity is 0.23 nm/%RH in the visible region and 0.34 nm/%RH in the NIR region. This proves the well-known concept that the sensitivity is increased at longer wavelengths^21^.

In addition, in Figs. S8 and S9 in the Supplementary Information section, the same test of Fig. 4 is performed for the sake of testing the repeatability of the sensor. The results prove that there are no perceptible changes in the performance of the sensor in terms of wavelength shifts.

Finally, in Table S1 there is a comparison of the sensitivities obtained in the different temperature and humidity tests performed, whilst a comparison with other sensors can be extracted from a couple of reviews on temperature and humidity sensors. Regarding temperature sensors, the sensitivity of the device proposed in this work improves the highest sensitivity of all sensors reported in^22^, which is 0.5 nm/°C, whereas optical humidity sensors present values that range from 1 pm/%RH to 1 nm/%RH^23^.

## Conclusions

The unique properties of LMR based sensors were used in a single platform towards the development of a dual parameter sensor. The sensor was based on a dually nanocoated planar waveguide that consists of one of the simplest and most cost-effective elements in a laboratory: a coverslip.

LMRs at typically generated at angles of incidence approaching 90°. This property permits to excite the coverslip waveguide by the edge and to deposit both faces of the coverslip. As a result, both faces and their corresponding LMRs can be used for sensing purposes. This is true because if either face is covered by a liquid with a specific refractive index or the coating refractive index or thickness is sensitive to some environmental parameter, the corresponding LMR will experience a wavelength shift according to the rules of design of LMR-based sensors^15^. Moreover, here it was demonstrated that both LMRs operate independently from each other.

This capability of dual parameter sensing based on the deposition of both faces of a planar waveguide is not possible with other planar waveguide based configurations, such as the well-known Kretschmann configuration, because one of the faces of the waveguide must be covered with an optical prism. Optical fibres also present the same limitation, because due to their dimensions it is very difficult to deposit different regions in the transversal axis of propagation of light.

Compared to optical fibre, the setup is very simple; it is only necessary to replace the coverslip with a new one to have a new sensor, whereas optical fibres require splicing and are easier to break. Moreover, the structure proposed here, contrary to multimode fiber, permits to isolate the TE and TM LMRs. It is true that D-shaped fibers can be used for the same purpose, but they are more expensive than multimode fiber and coverslips, and compared to D-shaped fibre, where it is necessary to use polarisation maintaining fibres or a polarisation system based on an in-line polariser and a polarisation controller, here it is just necessary to use a polariser in front of the coverslip.

Regarding the size of the system, the surface of the sensor is 18 × 18 mm and could be reduced even more, since LMRs can also be generated with smaller areas, whilst the rest of the setup is the same as for a multimode fiber. Finally, it must be mentioned that LMRs are very easy to tune in the optical spectrum by controlling the thickness of coating (some examples were shown in this work), which opens the path for deposition of coatings of different thickness in one or both sides of the coverslips, multiplying the number of LMRs and of parameters to detect.

Regarding the coatings, the materials used for generation of the LMRs may be sensitive to a specific parameter. However, it is normally a better approach to generate the LMR with a material that permits obtaining the LMR in the desired position, with an adequate depth, and to cover the LMR-generating material with another material that is specifically sensitive to the variable to be detected. In this work, it was shown that CuO presents the desirable property of satisfying the conditions for LMR generation in the visible and NIR range, with an additional PDMS layer for temperature sensing and an agarose layer for humidity sensing. The results have demonstrated that it is better to operate in the infrared range because the sensitivity is improved by a factor of 3.5 in the case of temperature and by a factor of 1.5 in the case of humidity.

Furthermore, the ability to detect two parameters with a single device opens the path for other applications, such as dual channel microfluidic systems for chemical sensors or biosensors, or the development of devices with more than two LMRs in the same spectral range, in view of the fact that, in terms of central wavelength, there is no influence among the resonances. To this purpose, it is necessary to apply nanopatterning techniques in each of the faces where the nanocoatings were deposited. In this way, the number of sensors on the same platform can be multiplied, generating a multi-parameter sensing platform, depending on the specific materials or nanopatterns deposited on each specific area of the coverslip. This novel sensing platform can be a valuable tool for the development of highly sensitive, compact and multi-parameter devices that can be used in many domains, such as environmental, gas, chemical sensors or biosensors. Similarly, different photonic devices, not only sensors, can be developed, such as filters, couplers, waveguides, etc. In other words, a countless number of new lines of research and applications can be opened with this simple multipurpose platform.

## Methods

### Experimental setup

Figure 1a shows the experimental setup used for the fabrication and characterisation of the dual parameter sensor. A multimode optical fibre FT200EMT with 200/225 µm core/cladding diameter (from ThorLabs, Bergkirchen, Germany), was placed in front of one of the lateral faces of a coverslip made of standard soda lime glass (from RS France, with dimensions 18 × 18 × 0.15 mm). The multimode fibre receives at one end light from a broadband source, HL-2000 (OceanOptics Inc., Largo, FL, USA), and transmits it to the coverslip at the other end. The output light transmitted by the opposite lateral face of the coverslip is received by another multimode optical fibre that is connected to an HR4000 spectrometer (OceanOptics Inc., Largo, FL, USA). In order to avoid interferometric phenomena, both lateral faces of the coverslip located in front of the multimode fires and the fibre tips were protected during deposition with a mask.

The planar waveguide was coated with CuO on the upper face, face 1, with a DC sputtering machine (K675XD from Quorum Technologies, Ltd., Lewes, UK). The parameters used in the experiment included an intensity of 75 mA and an argon partial pressure of 7 × 10^−2^ mbar. The CuO sputtering target (57 mm in diameter and 3 mm in thickness) was purchased from Loyal Target Technology Co, Beijing, China. The coverslip was positioned at a distance of 7 cm from the target. Once the first resonance was obtained, the coverslip was rotated and deposited on the lower face, face 2, with the same material and under the same conditions, but for a different period of time, in order to position the resonances corresponding to each face differently.

After the depositions, face 1 of the device was coated with PDMS (Sylgard 184) from Dow Corporate in order to make it sensitive to temperature. PDMS was prepared by mixing the elastomer base with the curing agent in 10:1 proportion and cured at 60 °C for 1 h. The device was made sensitive to humidity by coating face 2 with agarose using the method described in^24^, using an agarose concentration of 1% in water, applied using a spin-coating machine, model WS-650SZ-6NPP / LITE (from Laurell, North Wales, PA, USA), at 700 rpm and acceleration 2 rpm/sec for 3 min. Both the PDMS and the agarose coating were respectively characterized with an inverted microscope Buehler Met 89 and a scanning electron microscope (model UltraPlus FESEM from Carl Zeiss Inc.) with an in-lens detector at 3 kV and an aperture diameter of 30 μm).

In order to guarantee that both faces of the sensor were exposed to the same environmental conditions, a platform that held the waveguide upright was developed (see Fig. 1a). In addition, the setup included a linear polariser LPVIS050 from Thorlabs between the output of the optical fibre that launched light into the coverslip and the coverslip itself. This permitted excitation of the waveguide with linearly polarised light, which can be oriented horizontally or vertically to allow the separation of LMR_TE_ or LMR_TM_ respectively. In this way, resonances that were deeper and easier to track were obtained. During the CuO deposition this was not possible because the polariser can be damaged if it is introduced into the DC sputtering machine.

Regarding the reference signal, for the deposition of face 1, it was the amount of transmitted light that was measured at each wavelength before starting the deposition. However, for face 2 there were two options: to use the same reference signal used for face 1 or to take a new reference when the deposition of this face started. Depending on the selection, as shown in Figs. 1d and 2a, the resonances of one of the faces or both faces will be visualised.

### FIMMWAVE simulations

The optical field intensity distribution in the cross-section of the coverslip, coated with CuO on both face 1 and face 2, was simulated with the FIMMPROP, an integrated module of FIMMWAVE. The structure analysed consisted of a ridge waveguide segment with five layers: the coverslip, the two CuO coatings and the outer media. The thickness of the coverslip was 150 μm, whereas the thickness of the CuO coatings deposited on face 1 and face 2 of the coverslip was 40 nm and 60 nm respectively. These were the values that best fitted the experimental results. In addition, the finite difference method (FDM) was used for obtaining the effective indices and the optical field intensity of the modes.

Regarding the refractive index of the waveguide, since the coverslips were made of soda lime glass, the refractive index model of^25^ was used. In addition, for CuO the real part of the refractive index model of^26^ was used. This decision aimed at simplifying the analysis of the transition to guidance of coverslip modes in the coating. It is well-known that, with complex refractive index coatings, higher order modes experience this transition^27^, which complicates the explanation and interpretation of the results in a 150 μm thick waveguide with hundreds of modes.

### Temperature and humidity characterisation

For the characterisation of the dually nanocoated sensor, the same experimental setup of Fig. 1a was used, with the particularity that, in addition to the HR4000 operating in the range 400–1000 nm, another spectrometer for the region 900–1700 nm, NIRQuest 512 from OceanOptics, was used. In addition, the setup was installed in a climatic chamber type KMF-115 (from Binder, Tuttlingen, Germany).

Another major difference, compared to the deposition process, is that a new reference signal from a non-coated coverslip was taken, so as to avoid interferences due to the manipulation of the sensor when introducing it into the climate chamber, because for the deposition process the reference signal was the same obtained from the coverslip before starting the deposition. After that, the non-deposited coverslip is replaced by the deposited one, and the LMRs of face 1 and face 2 can be visualised in the spectrum. These LMRs were monitored during the temperature and humidity tests. In addition, the spectra were processed in real time using a least squares parabolic fit MATLAB algorithm, which permitted obtaining the central wavelengths of the LMRs along with colour maps, indicating the transmitted power at each different wavelength.

## Supplementary Information

Supplementary Information.
