# Supplementary material for: Dually nanocoated planar waveguides towards multi-parameter sensing
Source: Sci Rep. 2021 Feb 11;11:3669. doi: 10.1038/s41598-021-83324-8 (PMC7878508; doi:10.1038/s41598-021-83324-8)
Supplement: Supplementary file 1 — Supplementary Information. [file 41598_2021_83324_MOESM1_ESM.docx]

SUPPORTING INFORMATION to the manuscript

Dually nanocoated planar waveguides towards multiparameter sensing

Ismel Dominguez^1,2^, Ignacio Del Villar^1,3^, Omar Fuentes^1,2^, Jesus M. Corres^3^, Ignacio R. Matias^1^*

^1^Institute of Smart Cities, Public University of Navarre, 31006 Pamplona, Spain

^2^Department of Telecommunications and Electronics, Pinar del Río University, Pinar del Río CP 20100, Cuba

^3^Department of Electrical and Electronic Engineering, Public University of Navarre, 31006 Pamplona, Spain

[*natxo@unavarra.es](mailto:*natxo@unavarra.es)

FIMMWAVE Simulations

The optical field intensity distribution of the three first TM modes of the coverslip, TM_0_, TM_1_, TM_2_, is presented in **Figure S1** in the wavelength range from 480 to 530 nm. The mode TM_0_ experiences a transition to guidance in the CuO coating on the lower face of the coverslip. This transition occurs from 510 nm to shorter wavelengths, with a progressive concentration of the optical field intensity in the lower face of the coverslip (face 2). This agrees well will the optical spectrum of **Figure 2**, where the LMR_TM_ corresponding to the 60 nm CuO coating on face 2 of the coverslip is located at 450-500 nm. At the same time this occurs, the rest of modes experience a conversion to the immediate lower order mode, according to what was observed in previous works ^1,2^. For example, TM_1_ takes the shape of TM_0_ and TM_2_ the shape of TM_1_. In addition, an increase in the evanescent field is observed in all modes, especially at 510 nm.

In **Figure S2** the same analysis is performed for the TE modes in the wavelength range from 650 to 700 nm, where the guidance of a TE mode is observed in the upper face of the coverslip, which is coated with a 40 nm CuO thin film. The transition occurs this time for the second TE mode, TE_1_, from 680 m to shorter wavelengths, because TE_0_ is already guided at this wavelength range in the lower face of the coverslip (face 1). This agrees well with the wavelength range of LMR_TE_ in Figure 2: 650-700 nm. Regarding TE_2_, this mode takes the shape of TE_1_ at shorter wavelengths, and again an increase in the evanescent field is observed, this time especially at 680 nm.

Finally, **Figure S3** shows the evolution of TE modes in the wavelength range from 890 to 940 nm. Here a transition to guidance of the TE_0_ mode is observed from 920 to shorter wavelengths, with a progressive concentration of the optical field intensity in the lower face of the coverslip (face 2). This agrees well will the optical spectrum of **Figure 2**, where the LMR_TE_ corresponding to the 60 nm CuO coating on face 2 of the coverslip is located at 850-900 nm. At the same time this occurs, the rest of modes experience a conversion to the immediate lower order mode. TE_1_ takes the shape of TE_0_ and TE_2_ the shape of TE_1_. In addition, an increase in the evanescent field is observed in all modes, especially at 920 nm.


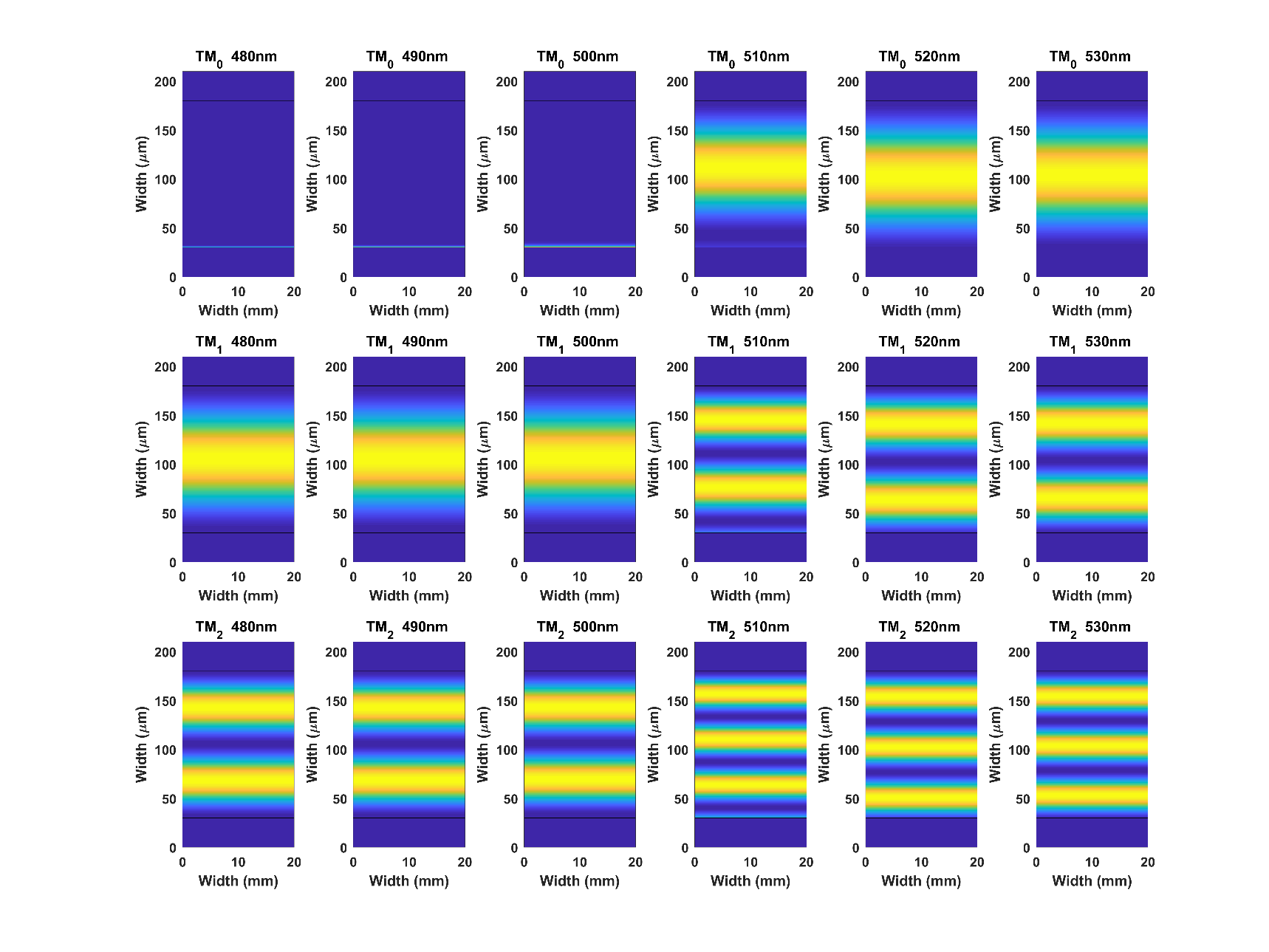


**Figure S1 | Optical field intensity distribution of TM_0_, TM_1_ and TM_2_ in the cross-section of a coverslip waveguide coated with CuO on both the upper and the lower face (wavelength range 480-530 nm).** TM_0_ experiences a transition to guidance in the CuO thin-film located at the lower face (face 2) of the coverslip for wavelengths shorter than 510 nm. The rest of modes, TM_1_ and TM_2_, turn into the shape of the immediate lower order mode (TM_0_ and TM_1_ respectively)_._


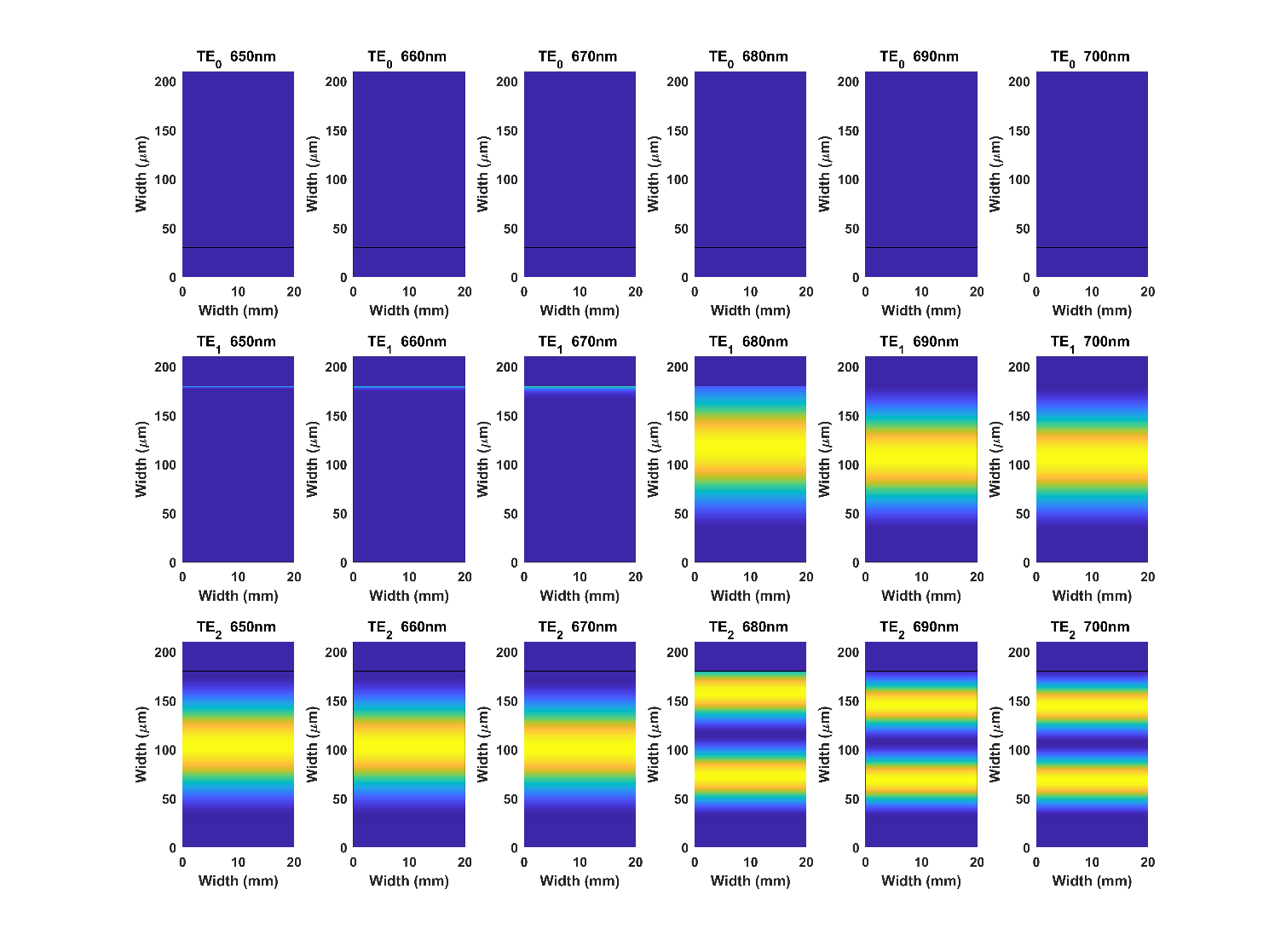


**Figure S2 | Optical field intensity distribution of TE_0_, TE_1_ and TE_2_ in the cross-section of a coverslip waveguide coated with CuO on both the upper and the lower face (wavelength range 650-700 nm).** TE_1_ experiences a transition to guidance in the CuO thin-film located at the upper face (face 1) of the coverslip for wavelengths shorter than 680 nm. TE_2_ turns into the shape of the immediate lower order mode: TE_1_.TE_0_ is already guided in the thin-film located at the lower part of the coverslip (the transition is observed in Fig. S3).


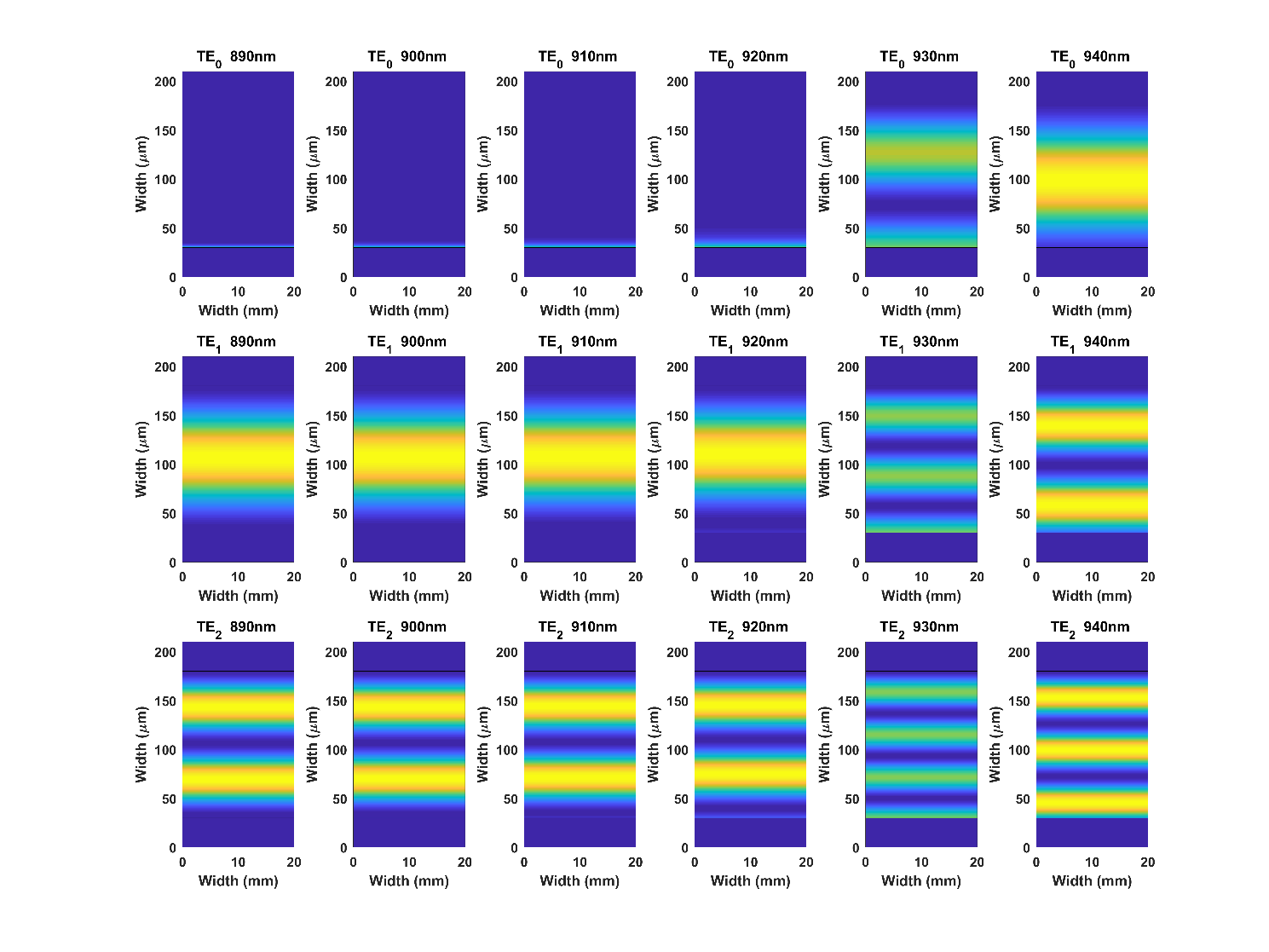


**Figure S3 | Optical field intensity distribution of TE_0_, TE_1_ and TE_2_ in the cross-section of a coverslip waveguide coated with CuO on both the upper and the lower face (wavelength range 890-940 nm).** TE_0_ experiences a transition to guidance in the CuO thin-film located at the lower face (face 2) of the coverslip for wavelengths shorter than 920 nm The rest of modes, TE_1_ and TE_2_, turn into the shape of the immediate lower order mode (TE_0_ and TE_1_ respectively)_._

In addition to the optical field intensity, the evolution of the effective index of the modes was analysed in Figure S4 in the wavelength range from 400 to 1000 nm, with transitions to guidance of modes TM_0_, TE_1_ and TE_0_ at the same wavelengths observed with the optical field analysis of Fig. S1-S3.


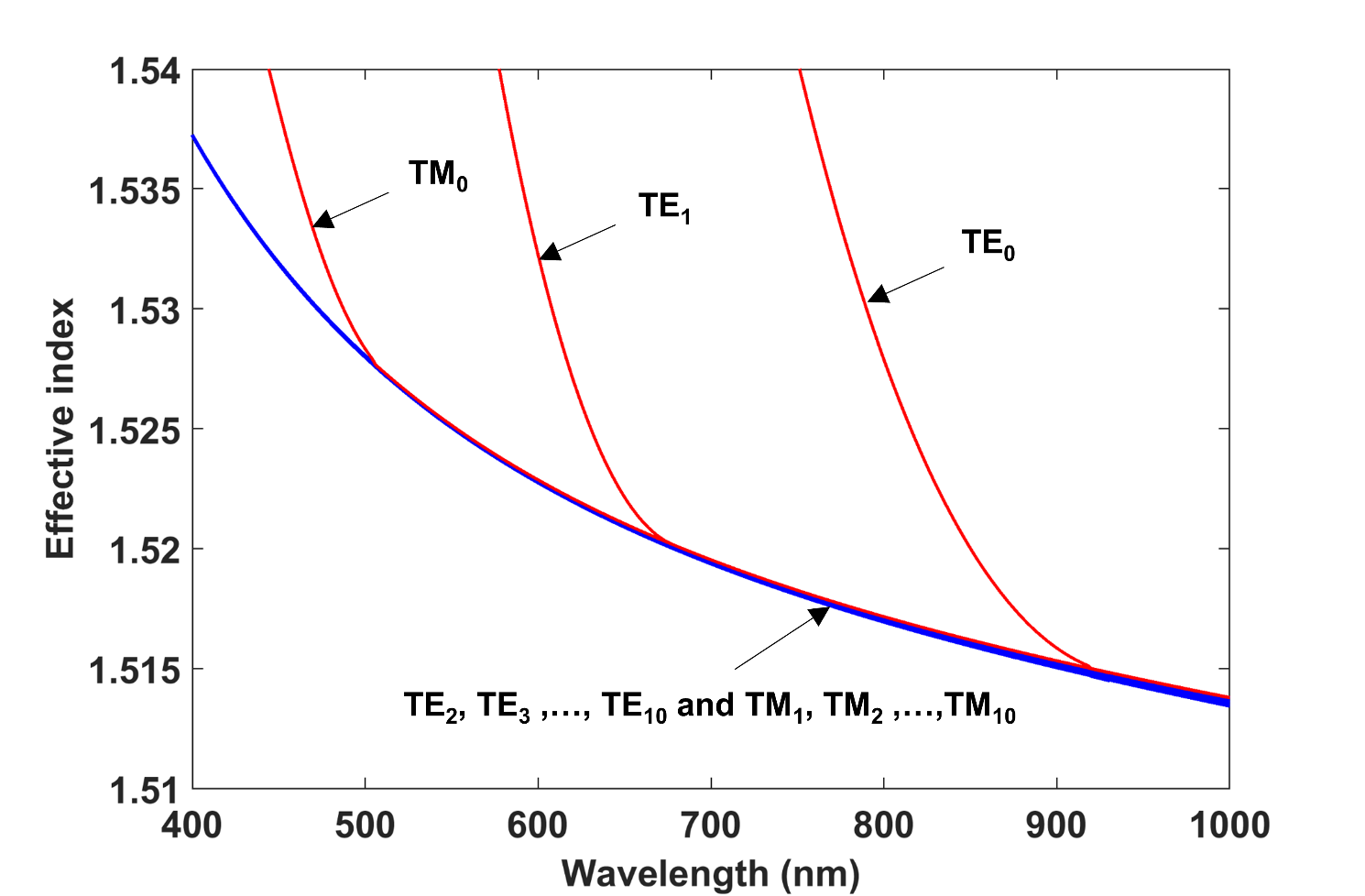


**Figure S4 | Effective indices of the first 10 TE and 10 TM modes.** Modes TM_0_, TE_1_ and TE_0_ experience a transition to guidance at the same wavelengths observed with the optical filed intensity analysis of Fig. S1-S3.

Temperature test

In Fig. S5 it is clear that only the LMRs induced by face 1, on which an additional PDMS layer was deposited, experienced a wavelength shift as a function of temperature. The average sensitivity in the temperature range from 30 to 70 ºC in the visible region is 0.35 nm/ºC, whereas in the infrared region it is 1.36 nm/ºC. In addition, the LMR recovers its original position after the temperature analysis.

Fig. S6 and S7 show a colour map of the transmitted power as a function of time and wavelength in the visible and in the NIR range, respectively. The central wavelengths of the LMRs are superimposed, and they follow the blue coloured regions that correspond with the LMR attenuation bands.

In addition, it must be highlighted that the colour map permits observing the evolution of the power as a function of temperature. In this sense, whilst in Fig. S6 there is no perceptible interaction between the two LMRs, in Fig. S7 the wavelength shift of the LMR located at shorter wavelengths, the one coated with PDMS, induces a decrease of the power of the LMR located at longer wavelengths. However, this variation is not responsible for any change in the wavelength of the LMR. In other words, the position of the LMR is independent of the presence of the other LMR, whilst this is not the case for the transmitted power.


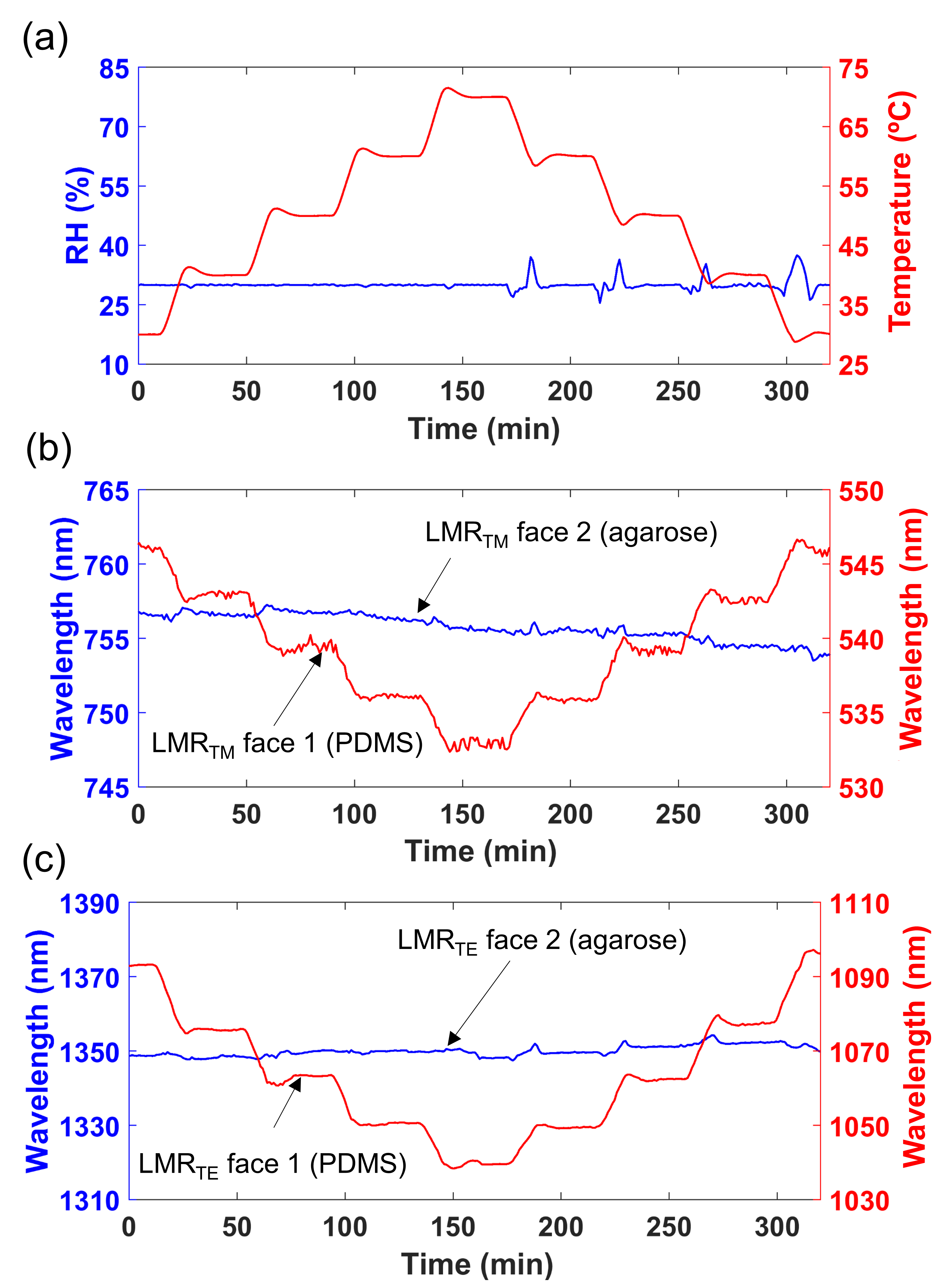


**Figure S5. | Temperature response of the electronic sensor compared to the optical sensor. (a)** Temperature values registered by the electronic sensor. **(b)** Wavelength shift in the visible range of the LMR_TM_ generated by face 2, coated with agarose, and of the LMR_TM_ generated by face 1, coated with PDMS. **(c)** Wavelength shift in the NIR range of the LMR_TE_ generated by face 2, coated with agarose, and of the LMR_TE_ generated by face 1, coated with PDMS.


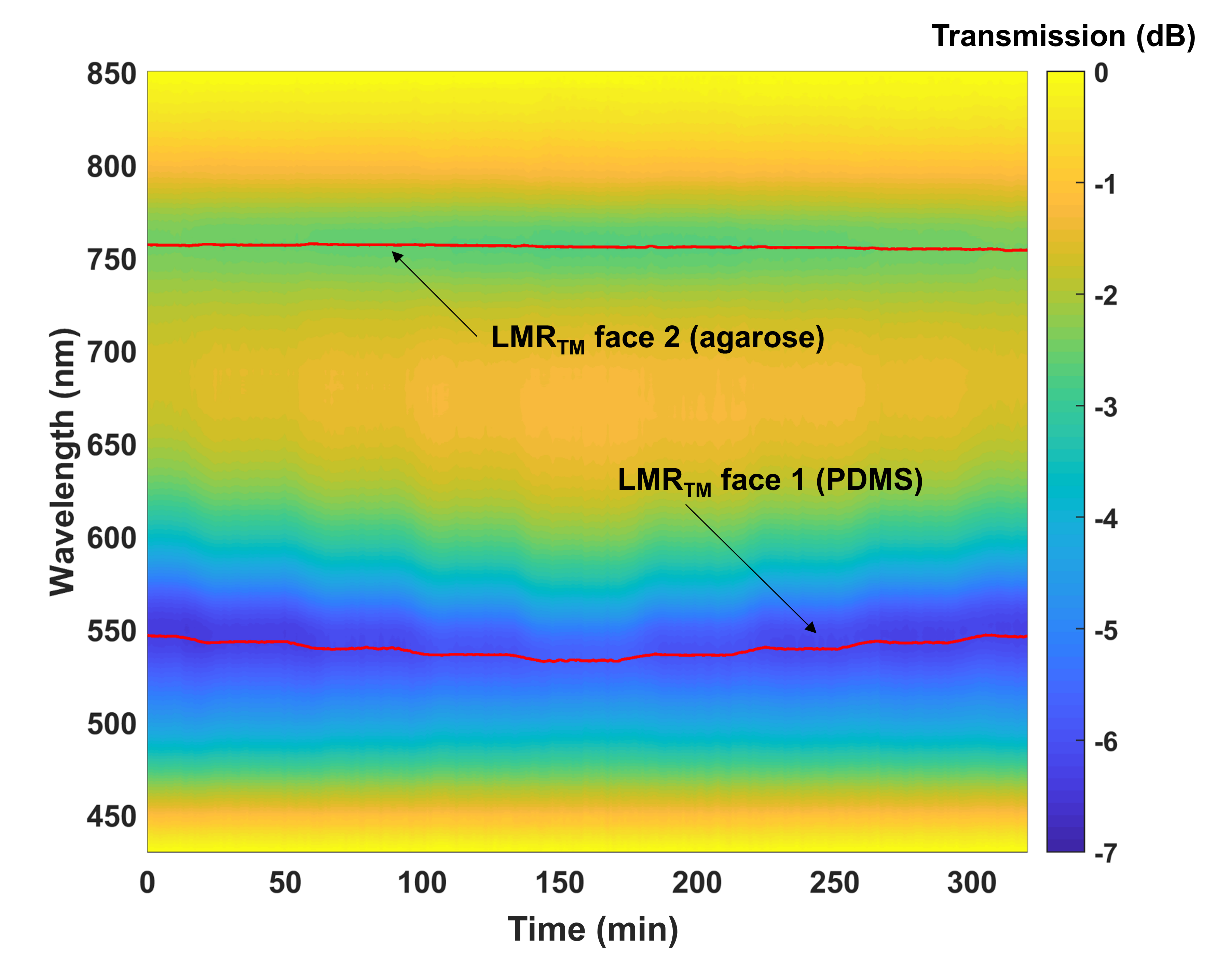


**Figure S6 | Spectral evolution in the visible range.** The central wavelengths of the LMR_TM_ induced by face 1 (PDMS) along with the LMR_TM_ induced by face 2 (agarose) are shown in red colour as a reference.


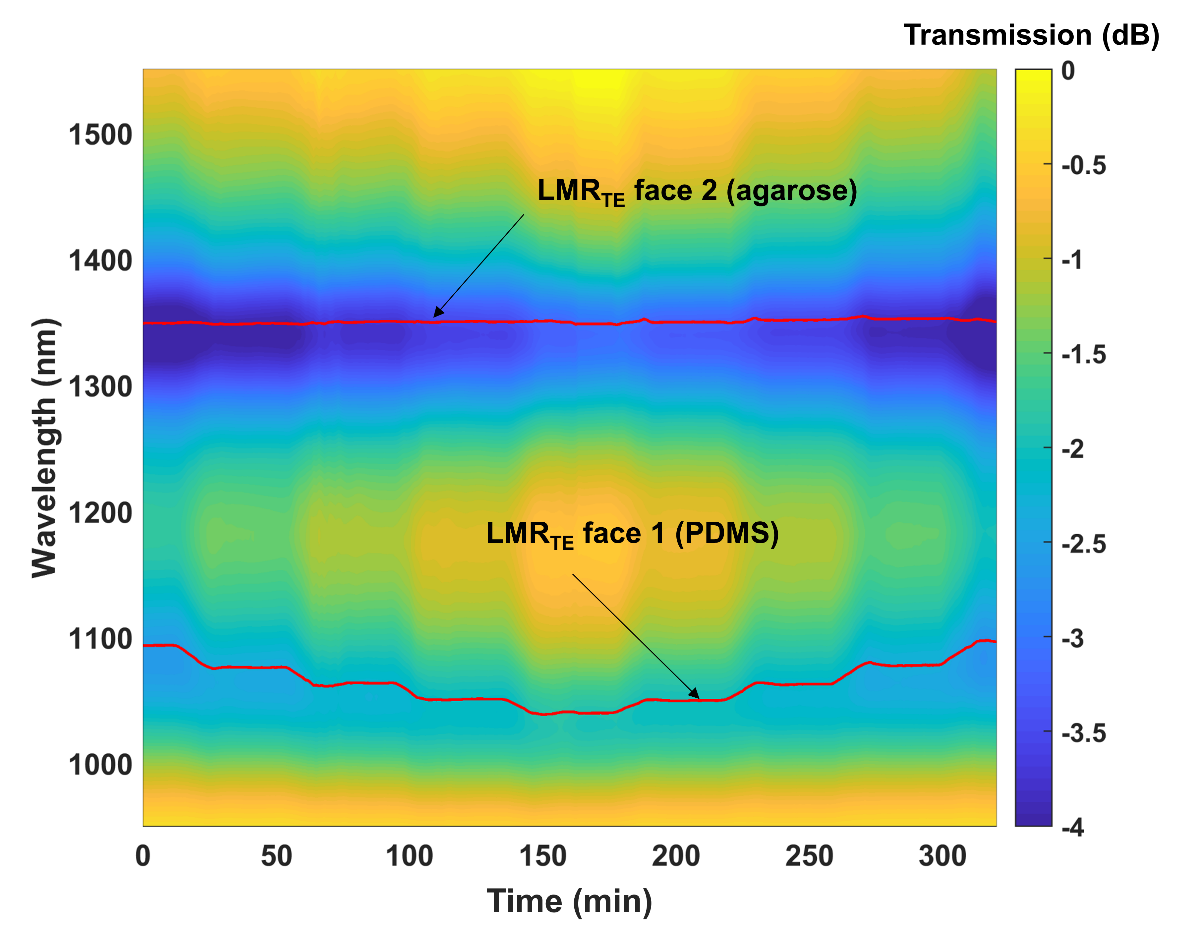


**Figure S7 | Spectral evolution in the NIR range.** The central wavelengths of the LMR_TM_ induced by face 1 (PDMS) along with the LMR_TM_ induced by face 2 (agarose) are shown in red colour as a reference.

Temperature and humidity test

Fig. S8 shows the colour map of the sensor in the infrared, for an analysis performed one week after the analysis of Fig. 4c. There is a change in the power values, but the central wavelengths of the resonances are very similar. Indeed, if Fig. S9 is compared with Fig. 4a, it is clear that there is a very small change in the results obtained. If the sensitivity is compared, it is observed that in Fig. S9 the sensitivity is 1.19 nm/ºC, whilst in Fig. 4a the sensitivity to temperature is 1.16 nm/ºC. Regarding humidity, in Fig. 4a a sensitivity of 0.35 nm/%RH is obtained whilst in Fig. 4a the sensitivity is 0.34 nm/%RH.

Finally, the sensitivities attained in all the experiments are shown in Table S1. There it can be observed that the temperature sensitivity in the NIR range experienced a perceptible variation from Test 1 to Test 2, whereas this is not the case if the results obtained in Test 2 and Test 3 are compared, in which the sensitivity to temperature in the NIR range is 1.16 nm/ºC and 1.19 nm/ºC, respectively. This stabilisation is also observed if the humidity sensitivity is compared: 0.34 vs 0.35 nm/%RH, which proves that after a first test, the sensor reaches a stabilisation in the results.


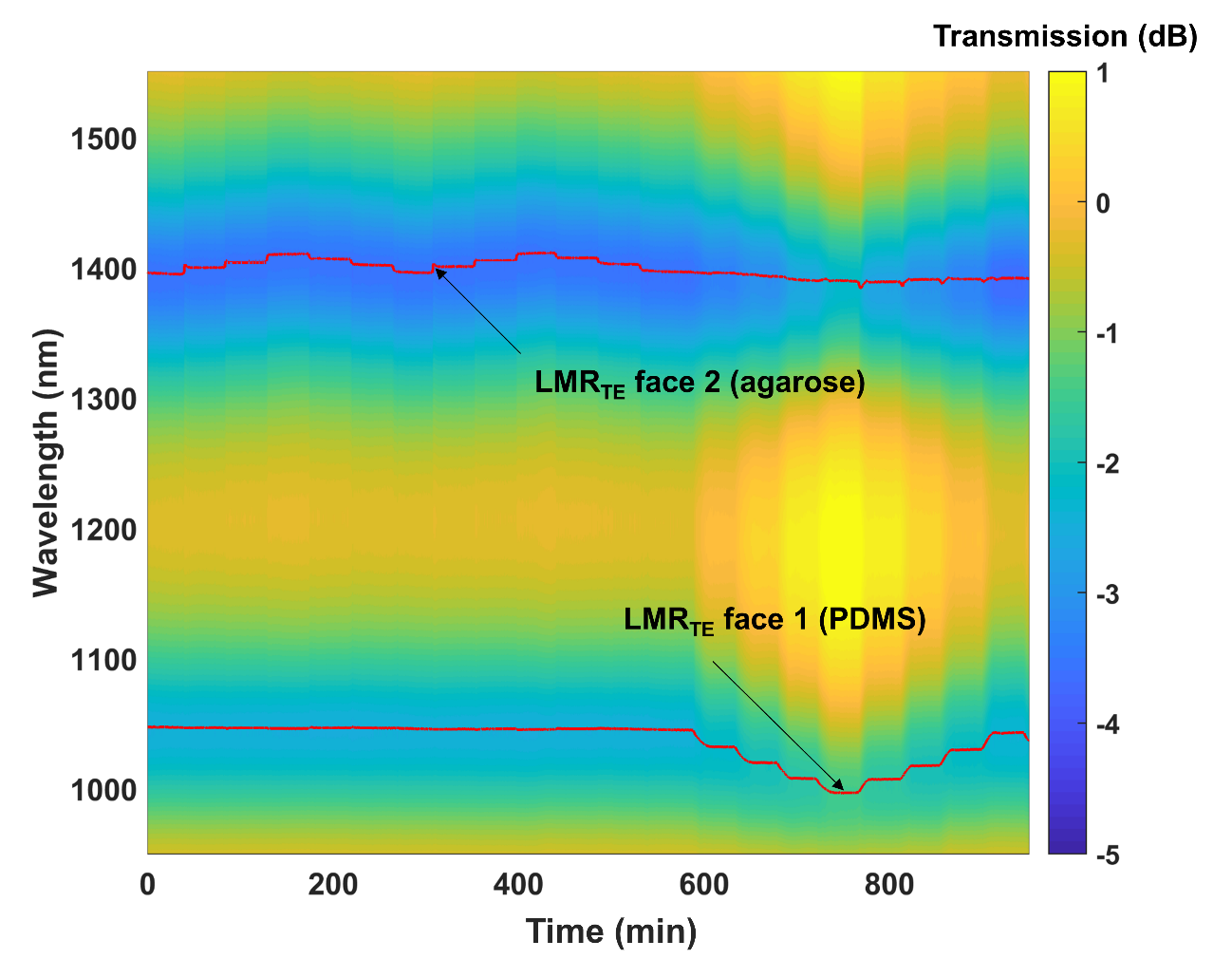


**Figure S8 | Spectral evolution in the NIR range.** Repeat of the experiment of Figure 4.


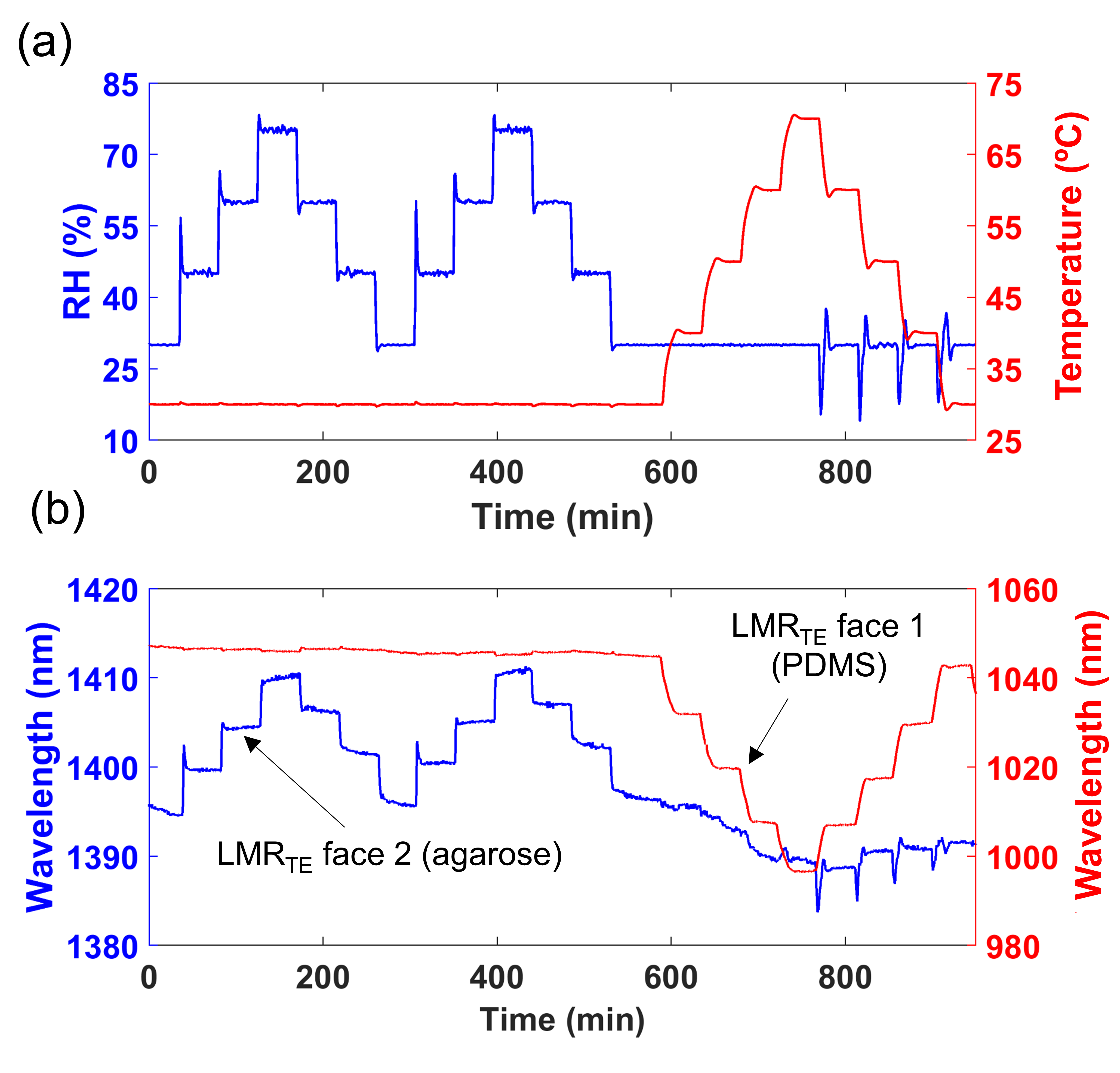


**Figure S9. | Humidity and temperature response of the electronic sensor compared to the optical sensor in the NIR range (repeat of the experiment of Figure 4). (a)** Humidity and temperature values registered by the electronic sensor. **(b)** Wavelength shift of the LMR_TE_ generated by face 2, coated with agarose, and of the LMR_TE_ generated by face 1, coated with PDMS.

**Table S1.** Temperature and humidity sensitivities in the visible and in the NIR range attained in the different tests

|  | Temperature Sensitivity | | RH Sensitivity | |
| --- | --- | --- | --- | --- |
|  | Vis (nm/ºC) | NIR (nm/%RH) | Vis (nm/ºC) | NIR (nm/%RH) |
| Test 1 | 0.35 | 1.36 |  |  |
| Test 2 | 0.34 | 1.16 | 0.23 | 0.34 |
| Test 3 |  | 1.19 |  | 0.35 |
